# Does *HOTAIR* expression level in the peripheral blood have veritably predictive/prognostic impact on breast cancer patients?

**DOI:** 10.1186/s12967-019-02158-1

**Published:** 2019-12-03

**Authors:** Vahid Ezzatizadeh, Hossein Mozdarani

**Affiliations:** Department of Medical Genetics, Faculty of Medical Sciences, Tarbiat Modares University, Tehran, Iran

**Keywords:** HOTAIR expression level, Biomarker, Breast cancer patients, Peripheral blood

## Abstract

As a peripheral blood biomarker, the crucial role of long non-coding RNA (lncRNA) HOTAIR has recently been suggested in many types of disorder. Among these reports, few investigations have indicated overexpression of HOTAIR transcript in the breast cancer patients’ peripheral blood. In this regard, we studied the potential impact of radiotherapy on the peripheral blood HOTAIR expression of different breast cancer patients. Curiously, no significant expression level of HOTAIR was determined in the breast cancer patients’ peripheral blood, before and after radiotherapy (10 Gy exposure). Deliberating these investigations raised some debates on the specificity of the utilized methods, the corresponding obtained findings and impact of HOTAIR expression on breast cancer predication, as a potential peripheral blood biomarker, which is discussed in this article.

## Introduction

HOX transcript antisense intergenic RNA (HOTAIR) is a long non-coding RNA (lncRNA) discovered at 2007. Despite the critical role of HOTAIR in the mammalians’ developmental processes, overexpression of this lncRNA has been reported to coordinate in several genetic abnormalities. Cancer is one of the multi-factorial diseases caused by genetic/epigenetic alterations. Current evidences have revealed the association of HOTAIR with progression, in addition to the prediction/prognosis of many different cancer types (including glioblastoma, liver, gastric, pancreatic, colorectal and cervical malignancies). In addition to the indicated malignancies, it has also been demonstrated that HOTAIR is up-regulated in various type of breast cancer tissues and cells. Obviously, expression level analysis of this lncRNA in the breast cancer patients’ peripheral blood could suggest a novel non-invasive biomarker on the basis of circulating RNA. Nevertheless, definitive conclusion of predictive/prognostic effect of HOTAIR expression in this type of cancer still remains challenging.

## Investigating the predictive/prognostic impact of HOTAIR on breast cancer patients

We have recently aimed to investigate regulation of HOTAIR in the whole peripheral blood (including circulating as well as white blood cell RNA) of breast cancer patients compared to normal individuals. The patients were diagnosed with unilateral invasive ductal carcinoma at the stages II and III (age mean of 58 years old). In terms of molecular pathology, they were ER^+^, PR^+^, Ki67^+^ and HER2^−^ (cut-off point: 10%) with no spread to the other parts of body, other than some lymph nodes. Additionally, potential effect of the radiotherapy on HOTAIR expression of these patients was evaluated, following the performance of ethic procedures. To determine HOTAIR expression level, total RNA was isolated from the whole blood samples, according to the manufacturer’s instruction (ParsTous, Iran). This procedure was followed by treatment of total RNA with DNase I (Sinacolne, Iran). cDNA was next prepared from the consistent amount of individual RNA samples, using a cDNA synthesis kit (ParsTous). Additionally, genomic DNA was isolated using the standard phenol/chloroform extraction method [1]. Fragment amplification PCR was quantitatively performed, using two specific HOTAIR primers: (Forward: 5′-TTCGCAGTGGAATGGAACGGAT-3′, Reverse: 5′-CGCCGGTCCTCCATTTCAGC-3′). This set of HOTAIR primers is able to amplify distinctive sizes of both genomic and transcriptomic products. For the individual samples, *GAPDH* expression level was also analysed by quantitative reverse-transcription PCR (qRT-PCR) to relatively determine HOTAIR expression level. Ultimately, genomic analysis was performed for some samples to validate the sensitivity and specificity of the HOTAIR primers.

**Fig. 1:**
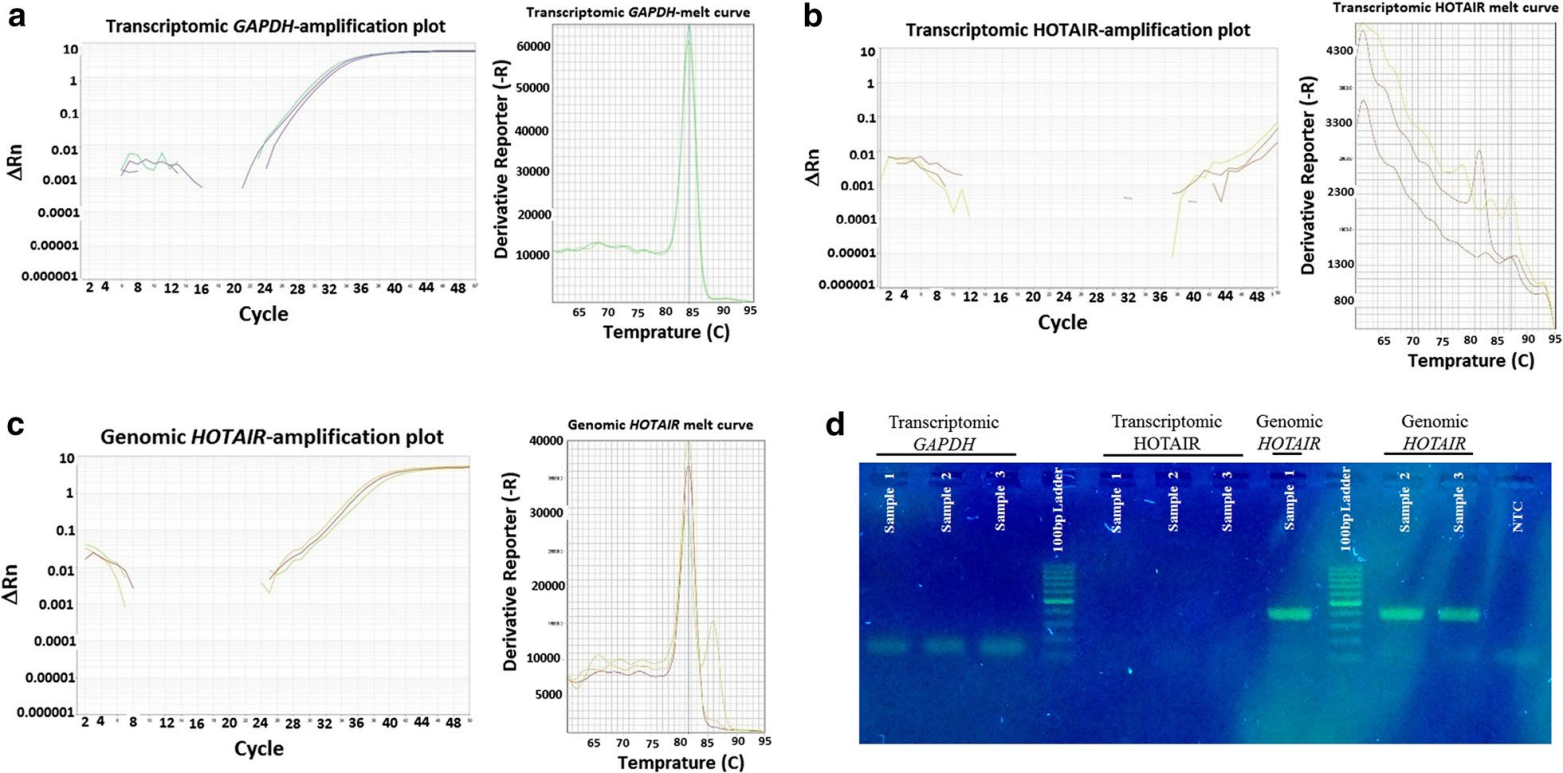
Schematic representation of the quantitative amplification plot and melt curve for **a***GAPDH* molecule in cDNA samples, **b** HOTAIR molecule in cDNA samples and **c***HOTAIR* molecule in genomic DNA samples. Image **d** also schematically shows qualitative fragment analyses of the indicated PCR products. The samples 1, 2 and 3 are respectively representative images of normal individual, breast cancer patients before and after radiotherapy (10 Gy). A specific fragment was determined for *GAPDH* (146 bp), while no fragment was generated from HOTAIR amplification in cDNA samples (potential cDNA fragment length: 197–201 bp depending on the different HOTAIR transcript variants; potential genomic fragment length: 389 bp). A specific fragment was also detected for *HOTAIR* (389 bp) in genomic DNA of all representative samples. NTC; Non-template control

Analysis of data obtained from the quantitative and qualitative PCR products showed expression level of HOTAIR in the peripheral blood of neither normal individual nor breast cancer patients before and after exposure to 10 Gy radiation (Fig. 1), whereas the quality of cDNA samples as well as sensitivity and specificity of the primers was appraised. Thus, evaluation of the expression level of *GAPDH* confirmed the quality and quantity of the individual prepared cDNA samples. Additionally, analysis of the genomic DNA validated sensitivity and specificity of the HOTAIR primers.

## Challenge

Apart from our recently obtained findings, four independent studies have thus far been performed to evaluate the predictive/prognostic impact of *HOTAIR* genomic or/and transcriptomic level(s) on the peripheral blood of breast cancer patients. In 2016, an article was published in the “Thoracic Cancer” journal suggesting overexpression of HOTAIR in circulating peripheral blood of breast cancer patients. Nevertheless, accuracy of this experiment could not be truthfully validated, regarding the wrongly highlighted primers utilized for the evaluation of HOTAIR expression level [2].

On April 2015, findings published in the “Journal of Translational Medicine” suggested up-regulation of HOTAIR expression in the breast cancer tissues and cell lines, through inhibiting miR-148 by estrogenic GPER signalling activity. This article also indicated overexpression of HOTAIR in the patients’ peripheral blood mononuclear cells [3]. Later, another investigation, published in the journal of “Cancer Biomarkers” on May 2018, also suggested positive association of circulating HOTAIR expression with tumour size, positive lymph node quantity and farther metastasis location [4]. Findings obtained from these studies raised the curiosity to navigate the cause(s) of this inconsistency on the mode of HOTAIR expression in the breast cancer patients’ circulating and peripheral blood mononuclear cells compared to the observed results in our experiment. Analysing the latter two experiments showed that similar to our experiment, the allocated HOTAIR primers could anneal both DNA and cDNA molecules, while no DNase treatment has been reported following the isolation of total RNA from the implicated samples. In this case, the data obtained from qRT-PCR would consequently represent combination of both genomic and transcriptomic HOTAIR levels and they could not realistically signify the HOTAIR circulatory or mononuclear cell RNA level in peripheral blood.

On the other hand, another original article was published on July 2015 in the “Breast Cancer Research and Treatment” journal suggesting *HOTAIR* circulatory DNA as a predictive/prognostic breast cancer biomarker. In this experiment, DNase I was utilized to evaluate RNA expression level of HOTAIR. Additionally, to evaluate genomic copy number of *HOTAIR*, RNA was not reverse transcribed. Findings showed no significant expression level of HOTAIR transcription, while *HOTAIR* circulatory DNA was almost duplicated in the serum of breast cancer patients compared to the normal individuals [5]. These findings are somehow in line with our finding, showing low expression level of HOTAIR. Curiously, analysing the data from the “Expression Atlas Biobank of European Bioinformatics Institute” also proposed no significant to very low HOTAIR transcript level in not only normal individuals’ but also breast cancer patients’ peripheral blood [6, 7].

## Conclusion

Current advances have introduced particular lncRNAs as potential molecular biomarkers utilized for prediction, diagnosis or prognosis of some disorders. HOTAIR up-regulation has also been determined in many types of malignant tissue and cell, including breast cancer. Despite some investigations suggesting the role of HOTAIR expression, as a potential peripheral blood molecular biomarker, in the prediction/prognosis of breast cancer, evidences obtained from the other studies propose that the obtained results from former experiments could carry some technical error. Thus, the significance might be prevailed due to the presence of *HOTAIR* circular DNA, but not a significant expression level of the corresponding RNA. This is in line with the data represented in the “Expression Atlas Biobank of European Bioinformatics Institute”. There is no doubt that further investigations would shed light on this discrepancy.

## Data Availability

The datasets used during current study are available from the corresponding author in reasonable request.

## Abbreviations

HOTAIR HOX: transcript antisense intergenic RNA
lncRNA: long non-coding RNA
qRT-PCR: quantitative reverse-transcription PCR
